# Supplementary material for: Best practices and practical strategies for co-designing virtual reality with Indigenous peoples: A scoping review protocol
Source: PLoS One. 2025 Jun 2;20(6):e0325111. doi: 10.1371/journal.pone.0325111 (PMC12129233; doi:10.1371/journal.pone.0325111)
Supplement: S1 Appendix — (DOCX) [file pone.0325111.s001.docx]

### **S1 Appendix.**

### **Search strategy for MEDLINE (EBSCOhost).**

| **Set** | **Search Terms** | **Search Modes** | **Results** |
| --- | --- | --- | --- |
| S1 | (MH "Indigenous Peoples+") | Proximity | 31,195 |
| S2 | TI ( Indigenous OR Aboriginal OR American N1 Indian OR "First Nations" OR “Native American*” OR “Alaska Natives” OR Aleut OR Andeans OR Anishinaabe OR Apache OR “Arctic Peoples” OR Assyrians OR Bedouins OR Berbers OR Cherokees OR Cheyenne OR Choctaws OR Circumpolar OR Cree OR Creek OR Eskimo OR Guarani OR Hopi OR Inuit OR Inuk OR Inupiat OR Inuvialuit OR “Irish Travelers” OR Kickapoo OR Kumeyaay OR Lakota OR Maasai OR Malays OR Maori OR Mapuche OR Maya OR Metis OR Mohawk OR “Native Hawaiians” OR Navajo OR Ojibwa OR Pima OR Sami OR Seminole OR Siletz OR “Tohono O'odham” OR “Torres Strait Islanders” OR Woodland OR Yupik OR Yupik OR Zapotec ) OR AB ( Indigenous OR Aboriginal OR American N1 Indian OR "First Nations" OR “Native American* ” OR “Alaska Natives” OR Aleut OR Andeans OR Anishinaabe OR Apache OR “Arctic Peoples” OR Assyrians OR “Australian races” OR Bedouins OR Berbers OR Cherokees OR Cheyenne OR Choctaws OR Circumpolar OR Cree OR Creek OR Eskimo OR Guarani OR Hopi OR Inuit OR Inuk OR Inupiat OR Inuvialuit OR “Irish Travelers” OR Kickapoo OR Kumeyaay OR Lakota OR Maasai OR Malays OR Maori OR Mapuche OR Maya OR Metis OR Mohawk OR “Native Hawaiians” OR Navajo OR Ojibwa OR Pima OR Sami OR Seminole OR Siletz OR “Tohono O'odham” OR “Torres Strait Islanders” OR Woodland OR Yupik OR Yupik OR Zapotec) | Proximity | 118,549 |
| S3 | S1 OR S2 | Proximity | 128,924 |
| S4 | (MH "Virtual Reality+") | Proximity | 8,138 |
| S5 | TI (virtual W3 reality OR mixed W3 reality OR VR OR avatar) OR AB (virtual W3 reality OR mixed W3 reality OR VR OR avatar) | Proximity | 30,189 |
| S6 | S4 OR S5 | Proximity | 31,472 |
| S7 | TI (participatory N1 design OR codesign* OR co-design* OR human-cent#red design* OR user-cent#red design* OR patient-cent#red design* OR interaction design* OR collaborat* design*) OR AB (participatory N1 design* OR codesign* OR co-design* OR human-cent#red design* OR user-cent#red design* OR patient-cent#red design* OR interaction design* OR collaborat* design*) | Proximity | 25,342 |
| S8 | S3 AND S6 AND S7 | Proximity | 3 |

### Search conducted on March 8, 2025
