# Supplementary material for: Best practices and practical strategies for co-designing virtual reality with Indigenous peoples: A scoping review protocol
Source: PLoS One. 2025 Jun 2;20(6):e0325111. doi: 10.1371/journal.pone.0325111 (PMC12129233; doi:10.1371/journal.pone.0325111)
Supplement: S2 Appendix — (DOCX) [file pone.0325111.s002.docx]

### **S2 Appendix.**

### **Data extraction instrument.**

| Articles  (countries) | Participants  (Sample size) | Study Designs | Indigenous people | | VR | Practical Strategies | | Best Practices |
| --- | --- | --- | --- | --- | --- | --- | --- | --- |
| Author, year |  |  |  |  | | |  |  |
| Author, year  Author, year  Author, year  Author, year  …... |  |  |  |  | | |  |  |
