# Supplementary material for: Best practices and practical strategies for co-designing virtual reality with Indigenous peoples: A scoping review protocol
Source: PLoS One. 2025 Jun 2;20(6):e0325111. doi: 10.1371/journal.pone.0325111 (PMC12129233; doi:10.1371/journal.pone.0325111)
Supplement: Attachment — Submitted filename: Response to Reviewers.docx [file pone.0325111.s006.docx]

April 28, 2025

Dr. Diogo Guedes Vidal

Academic Editor

PLOS ONE

**Practical Strategies and Best Practices in Co-Designing Virtual Reality with Indigenous**

**Peoples and Communities: A Scoping Review Protocol [PONE-D-25-12586] –– Response to Reviewers**

Dear Dr. Vidal,

Thank you for your thoughtful editorial review and for the opportunity to revise our manuscript. We sincerely appreciate the feedback provided to improve the clarity, transparency, and alignment of our protocol with PLOS ONE’s submission standards. Here, we provide a point-by-point response to the editorial and reviewer comments, along with the actions we have taken in the revised manuscript.

| **Academic Editor** | |
| --- | --- |
| **Comment** | **Response & Action** |
| 1. Please ensure that your manuscript meets PLOS ONE’s style requirements, including those for file naming. The PLOS ONE style templates can be found at <https://journals.plos.org/plosone/s/file?id=wjVg/PLOSOne_formatting_sample_main_body.pdf> and <https://journals.plos.org/plosone/s/file?id=ba62/PLOSOne_formatting_sample_title_authors_affiliations.pdf>. | We have revised our manuscript to align with the PLOS ONE formatting guidelines, including style and file naming conventions, as outlined in the templates provided. All files have been renamed and formatted accordingly. |
| 2. Please include a complete copy of PLOS’ questionnaire on inclusivity in global research in your revised manuscript. Our policy for research in this area aims to improve transparency in the reporting of research performed outside of researchers’ own country or community. The policy applies to researchers who have travelled to a different country to conduct research, research with Indigenous populations or their lands, and research on cultural artefacts. The questionnaire can also be requested at the journal’s discretion for any other submissions, even if these conditions are not met. Please find more information on the policy and a link to download a blank copy of the questionnaire here: <https://journals.plos.org/plosone/s/best-practices-in-research-reporting>. Please upload a completed version of your questionnaire as Supporting Information when you resubmit your manuscript. | We have completed the PLOS questionnaire on inclusivity in global research and uploaded it as Supporting Information (S4 Appendix) as per journal policy. We appreciate this important step in promoting transparency and ethical engagement in Indigenous research contexts. |
| 3. Please provide a complete Data Availability Statement in the submission form, ensuring you include all necessary access information or a reason for why you are unable to make your data freely accessible. If your research concerns only data provided within your submission, please write “All data are in the manuscript and/or supporting information files” as your Data Availability Statement. | We have included the following Data Availability Statement in the submission form: “All relevant data from this study will be made available upon study completion.” |
| 4. Please review your reference list to ensure that it is complete and correct. If you have cited papers that have been retracted, please include the rationale for doing so in the manuscript text, or remove these references and replace them with relevant current references. Any changes to the reference list should be mentioned in the rebuttal letter that accompanies your revised manuscript. If you need to cite a retracted article, indicate the article’s retracted status in the References list and also include a citation and full reference for the retraction notice. | Two authors (JW and KY) have thoroughly reviewed the reference list to ensure accuracy and completeness. We confirm that as of April 28, 2025, no retracted articles have been cited. |
| **Reviewer 1** | |
| **Comment** | **Response & Action** |
| 1. In terms of method, there is a lack of the following items required by Plos One in the study planning: hypothesis, sample size calculation, type of data and statistical analyses to be used and where and when the data will be made available. | Thank you for your comment. As this is a protocol for a scoping review, it does not involve primary data collection or hypothesis testing and therefore does not require a sample size calculation or statistical analysis plan. Scoping reviews are designed to “determine the scope or coverage of a body of literature on a given topic and give clear indication of the volume of literature and studies available as well as an overview (broad or detailed) of its focus” (Munn et al., 2018). Our data availability statement will be submitted through the submission system.  Reference:  Munn, Z., Peters, M. D. J., Stern, C., Tufanaru, C., McArthur, A., & Aromataris, E. (2018). Systematic review or scoping review? Guidance for authors when choosing between a systematic or scoping review approach. *BMC Medical Research Methodology*, *18*(1), 143. <https://doi.org/10.1186/s12874-018-0611-x> |
| 2. Line 142: What will be the analysis period? Please, include. | Thank you for the comment. As this is a scoping review, we interpret “analysis period” to refer to the expected timeframe during which study screening, data extraction, and synthesis will occur. We have added the following sentence to our manuscript to clarify this timeline:  **Line 135:** “**Screening, data extraction, and analysis will be conducted between March and June 2025.**” |
| 3. Line: 189: I suggest improving the wording by stating that these will be published articles. The word “available” gives the impression that only open access articles will be analyzed. | We agree with this suggestion and have replaced “publicly available” with “published and unpublished” to better reflect the scope of our review. The revised sentence now reads:  **Line 203:** “As this scoping review draws on **published and unpublished** articles, research ethics board approval is not required. The review **will be submitted** to a **peer-reviewed,** open-access journal to ensure broad accessibility.” |
